# Numerical simulation of the free surface and water inflow of a slope, considering the nonlinear flow properties of gravel layers: a case study

**DOI:** 10.1098/rsos.172109

**Published:** 2018-02-28

**Authors:** Bin Yang, Tianhong Yang, Zenghe Xu, Honglei Liu, Wenhao Shi, Xin Yang

**Affiliations:** 1 Center of Rock Instability and Seismicity Research, School of Resources and Civil Engineering, Northeastern University, Shenyang, Liaoning 110819, People's Republic of China; 2 Key Laboratory of Ministry of Education on Safe Mining of Deep Metal Mines, Northeastern University, Shenyang, Liaoning 110819, People's Republic of China

**Keywords:** slope, gravel layers, Forchheimer flow, non-Darcy coefficient, water inflow, free surface

## Abstract

Groundwater is an important factor of slope stability, and 90% of slope failures are related to the influence of groundwater. In the past, free surface calculations and the prediction of water inflow were based on Darcy's law. However, Darcy's law for steady fluid flow is a special case of non-Darcy flow, and many types of non-Darcy flows occur in practical engineering applications. In this paper, based on the experimental results of laboratory water seepage tests, the seepage state of each soil layer in the open-pit slope of the Yanshan Iron Mine, China, were determined, and the seepage parameters were obtained. The seepage behaviour in the silt layer, fine sand layer, silty clay layer and gravelly clay layer followed the traditional Darcy law, while the gravel layers showed clear nonlinear characteristics. The permeability increases exponentially and the non-Darcy coefficient decreases exponentially with an increase in porosity, and the relation among the permeability, the porosity and the non-Darcy coefficient is investigated. A coupled mathematical model is established for two flow fields, on the basis of Darcy flow in the low-permeability layers and Forchheimer flow in the high-permeability layers. In addition, the effect of the seepage in the slope on the transition from Darcy flow to Forchheimer flow was considered. Then, a numerical simulation was conducted by using finite-element software (FELAC 2.2). The results indicate that the free surface calculated by the Darcy–Forchheimer model is in good agreement with the *in situ* measurements; however, there is an evident deviation of the simulation results from the measured data when the Darcy model is used. Through a parameter sensitivity analysis of the gravel layers, it can be found that the height of the overflow point and the water inflow calculated by the Darcy–Forchheimer model are consistently less than those of the Darcy model, and the discrepancy between these two models increases as the permeability increases. The necessity of adopting the Darcy–Forchheimer model was explained. The Darcy–Forchheimer model would be applicable in slope engineering applications with highly permeable rock.

## 1. Introduction

In China, from 1949 to 2011, even the most conservative estimates of the deaths from landslides exceed 25 000 people, which was a death toll of more than 400 people each year, and the average annual pecuniary loss was $50 million dollars [1]. Groundwater is one of the main factors causing instability and sliding failure of the soil slope. A total of 90% of slope failures, for either natural slopes or artificial slopes, are related to groundwater [2]: more specifically, porewater pressures and seepage forces. It can be seen that understanding the seepage mechanism of slopes is necessary to prevent the occurrence of landslides.

The effect of water on the slope stability can be seen by considering the slope with different positions of the free surface [3]. Slope failures associated with the water level fluctuations were reported by many researchers, such as Lane [3], Jappelli & Musso [4], Shi & Zheng. [5], Jia *et al*. [6], Wang *et al*. [7] and Lu *et al*. [8]. The position of the free surface has a great effect on the slope stability; the stability safety coefficient of a soil slope gradually decreases with the continuous increase in the free surface area, until slope sliding occurs [9]. When the velocity of flowing reservoir water is uniform, a simplified formula, based on Boussinesq's theory, can be used to calculate the location of the free surface; a numerical analysis indicated that when the free surface is one-third of the slip body, the stability coefficient of the slope is minimal [5]. To investigate the influence of the drawdown on the slope stability and pore water pressure, Wang *et al*. [7] performed some laboratory physical model tests. Lu *et al.* [8] adopted an appropriate method to evaluate slope stability during earthquakes, including the effect of the groundwater level; this method extended the scheme of the limit equilibrium method. The free surface fluctuations may induce the change in the pore water pressure of the soil slope. Therefore, calculating the free surface of the soil slope is very useful to analyse the stability of the slope.

Moreover, it is well known that the water inflow of a slope has a very strong influence on the slope stability. Some fine particles are washed away due to the effect of water seepage, which is a main cause of mass loss. Hu *et al.* [10] adopted a group of orthogonal tests to investigate the influence of water capacity on sand-soil slope stability. To study the water inflow of an open-pit slope of the Ekou Iron Mine in China, Liu *et al.* [11] built a numerical seepage model based on GeoStudio and AutoCAD-3Dmine. Slope stability analyses and supporting studies under the condition of a larger foundation pit water inflow have been studied by Ma [12].

The above research all assumes that the water seepage in the slope obeys Darcy's law. However, the slope of the Yanshan Iron Mine contains two high-porosity gravel layers, which are very sensitive to the flow of groundwater. The results of laboratory experiments indicate that the fluid migration in these gravel layers follows non-Darcy flow and not Darcy's law. Li [13] also performed similar studies.

Until now, many studies on the Darcy fluid flow in this slope have been carried out, but the analysis of the non-Darcy flow of the slope is less reported in relevant publications. According to different theories and methods, including Darcy theory and non-Darcy theory, the results of these studies are sometimes very different. To investigate this problem, this paper first expounds the general situation of the Yanshan Iron Mine in §2. In addition, this paper presents experimental equipment used to test the specimens of soil which form different layers of the Yanshan Iron Mine slope, and the seepage properties, including the non-Darcy coefficient, are determined from measurements and calculations, as described in §3. A coupled model that includes two flow fields is first established based on the theory of conservation of mass and pressure balance; this model couples the equations of the Darcy flow field and the Forchheimer field, as described in §4. Then, the numerical results, the effect of the porosity of the gravel layers on the water inflow and the free surface are described in §5. Finally, §6 provides conclusions and discusses the implication of these findings.

## 2. Geology of the study area

**Figure 1.**
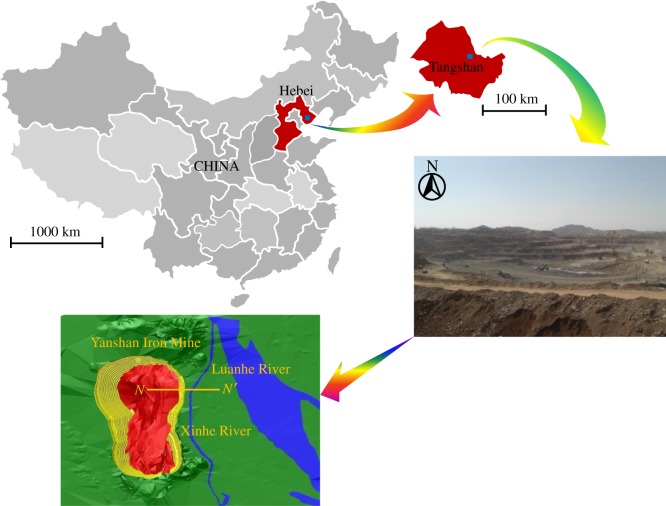
Location map of the study area.

**Figure 2.**
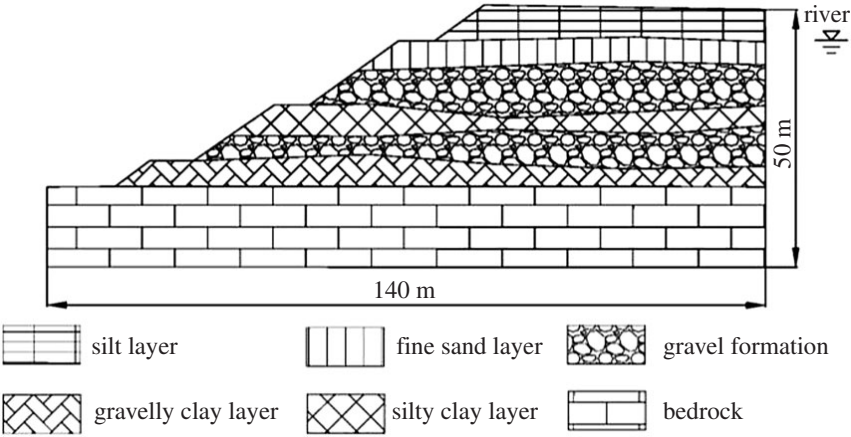
The geologic section of N–N′.

**Figure 3.**
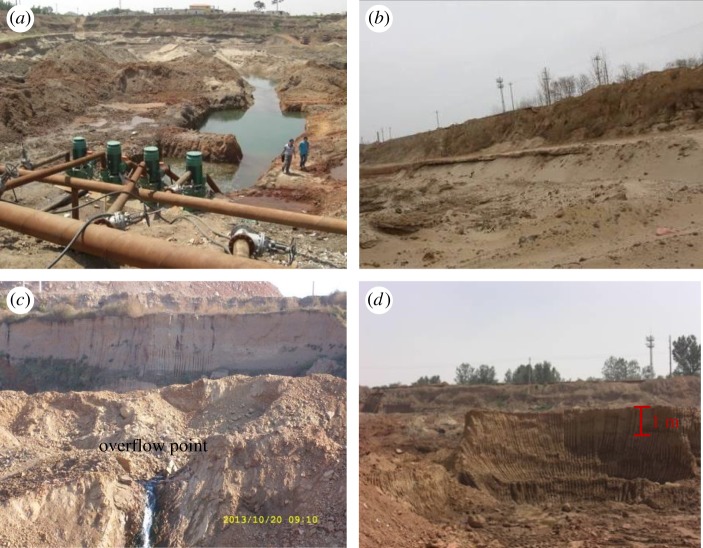
The geological exploration photos (*a*) drainage system, (*b*) fine sand layer, (*c*) location of the overflow point in the gravel layer and (*d*) silty clay layer.

The eastern slope of the open-pit Yanshan Iron Mine in Tangshan City, Hebei Province, China, was chosen as the study area, as shown in figure 1. Its specific location is 39.6° N, 118.8° E. According to the engineering geological investigation data, the eastern slope of the open-pit Yanshan Iron Mine is near the water conveyance canal of the Xinhe River, which is 61 m from the final mining boundary of the open pit. The average amount of water flowing down the river from 1963 to 2003 was 31 m^3^ s^−1^ and varied between 0.24 and 155.05 m^3^ s^−1^ in the most recent five years of that period; this variation was controlled by human activity. The velocity of the water flow in the gravel formation has been measured at 2.3 mm s^−1^. As you can see in figure 2, from top to bottom, the first, second, fourth, sixth and seventh layers of the slope are silt, fine sand, silty clay, gravelly clay and bedrock, respectively, which have low permeabilities. However, the third and fifth layers are gravel, which have high permeabilities and are water-abundant. The boundary of the gravel layers intersects the base of the Xinhe River riverbed and is dipping toward the bottom of the open pit. The groundwater for the slope is mainly sourced from the Xinhe River, which can lead to high water levels. The gravel layers and the fine sand layer are dipping to the southwest, and the top of the bedrock includes the old waterway of the Luanhe River. From the slope profile, it can be found that the permeable layers form the main percolation path, and the Xinhe River serves as the main source of constant head for the groundwater of the slope. Groundwater flows into the open pit through the gravel layers in large volumes and causes high water levels. The geological exploration photos (figure 3), show the location of the overflow point.

## 3. Experimental details

### 3.1. Experimental equipment

**Figure 4.**
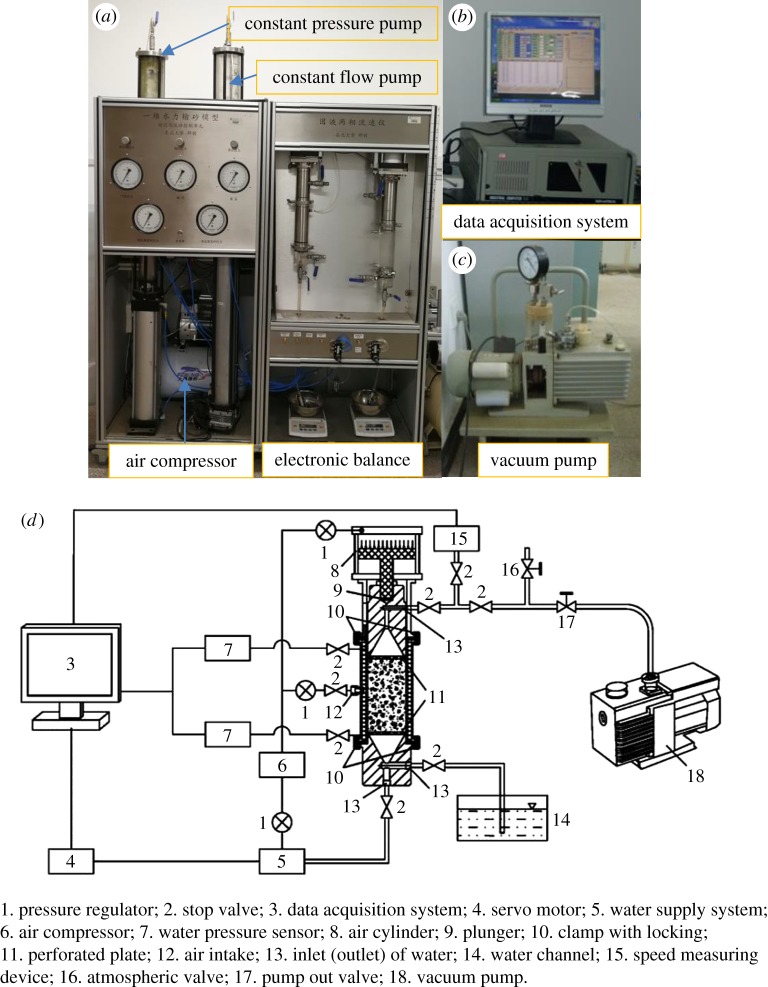
Experimental apparatus: (*a*) conveyance and flow control system and solid–liquid two-phase flow meter; (*b*) data acquisition system; (*c*) vacuum pump; and (*d*) experimental schematic diagram.

This experiment used a custom-built apparatus to model high-velocity seepage in a porous medium at Northeastern University, China. The conveyance and flow control system, solid–liquid two-phase flow meter, data acquisition system, saturation device and back-pressure device are the five major components in the testing system, as shown in figure 4. In addition, figure 4*d* illustrates the system connections and principle: the water supply system (5) consists of a constant pressure water supply and constant flow water supply. The volume of the constant pressure pump is 12 l, and it can provide water pressure at 2 MPa to the upstream of the sample. The maximum flow during the tests is 25 l min^−1^. In addition, the constant flow pump can provide a stable water flow at 0–3 l min^−1^. The air compressor (6), pressure regulator (1), air cylinder (8) and plunger (9) are used to apply the axial loading pressure. The porous plates (11) can ensure that the fluid flows evenly and protects the structure of the porous media so that it is not destroyed under high water pressures. The water pressure on the upstream and downstream ends of the sample are measured by pressure sensor (7) (Q/JL015-2009) in the range of 0 to 2.38 MPa ± 0.1% FS. The test tube is made of organic glass with a diameter of 120 mm and strength of 2 MPa, allowing visualization of the testing process. To avoid the effects of air on the experiments, the following measures were taken. Before starting the experiments, the vacuum pump (18) was firstly used to remove the air from the inside of the sample. Then, the water was slowly passed through the tube from the bottom to the top, until the sample saturation was above 95%. In addition, the atmospheric valve (16) can be used to discharge air if there is air trapped in the tube. In the flow test, if the outlet (13) is directly open to the atmosphere, air fluctuation often causes fluid instability and the veracity of the measurement results cannot be ensured, even when using high-precision sensors. To avoid the outlet effects, back pressure must be applied at the outlet. The speed measuring device (15) includes an electronic balance (TCL 12000), in the range of 0 to 12 000 g ± 0.1% FS, and large beakers. Data collection and analysis are performed by the data acquisition system (3), and the sampling interval is 3 s.

### 3.2. Experimental materials

**Figure 5.**
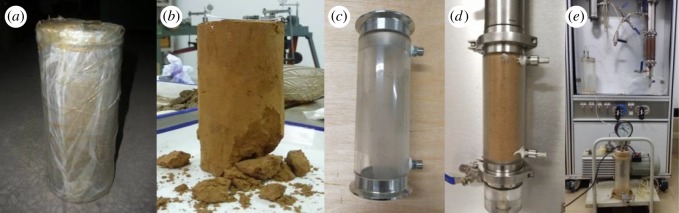
The preparation process before experimentation (*a*) sample package, (*b*) silt, (*c*) cylinder tube, (*d*) test sample, (*e*) vacuuming and saturating the sample.

The soil specimens used in the tests were taken from the eastern slope of the Yanshan Iron Mine, China, and were prepared in the laboratory. The soil columns were sampled from a borehole. The diameter of all the soil columns is 120 mm, which is equal to the internal diameter of the test cylinder. To eliminate the boundary effect, before the tests, the inside of the test cylinder was coated with a thin layer of petroleum jelly. A vacuum pump was used to remove any air bubbles from the test cylinder. The measurements were carried out under a stable flow. The main method to experimentally measure the flow rate was the weighing method. Figure 5 shows the preparation process before the experiments were conducted.

### 3.3. Water flow experiments

**Figure 6.**
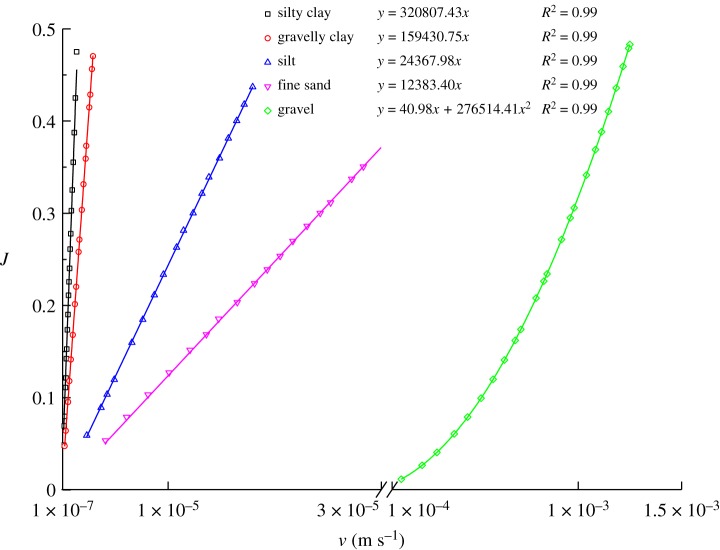
Relation between the hydraulic gradient and velocity.

To evaluate the permeability of each layer, all experimental data and the regression relationships between the hydraulic gradient and flow velocity were analysed and are depicted in figure 6. For the silt, fine sand, silty clay and gravelly clay layers, the relationships are linear, and the flow behaviour is Darcy flow. However, these relationships are nonlinear for the gravel layers; therefore, the flow behaviour is considered nonlinear flow [14].

The one-dimensional Darcy equation can be written as

3.1 $$J=\frac{\mu }{\rho gk}v,$$

where *J* is the hydraulic gradient, *μ* is the dynamic viscosity, *k* is the permeability, *g* is the gravitational acceleration, *ρ* is the fluid density and *v* is the superficial velocity.

The non-Darcy groundwater seepage problem is very complicated, and theoretical studies of this problem still use the Darcy flow theory. Until now, there was no formula that could describe this flow characteristic more accurately [15]. Through numerous tests, the empirical formulae of nonlinear flow were obtained. There are two basic forms: the Izbash equation and the Forchheimer equation [16]. The Forchheimer equation has a clear physical meaning and theoretical basis and is, therefore, widely used [17–19], the Forchheimer equation can be written as

3.2 $$\nabla p=\frac{\mu }{k}v+\rho \beta v^{2},$$

where ∇p is the pressure gradient and *β* is the non-Darcy coefficient.

To measure *k* and *β*, ∇p/μν is denoted as *y* and *ρv*/*µ* is denoted as *x*. Equation (3.2) then simplifies to

3.3 $$y=\frac{1}{k}+\beta x.$$

According to the laboratory experimental data, in the *x*–*y* coordinate system, a straight line is defined with slope *β* and *y*-axis intercept 1/*k*.

**Figure 7.**
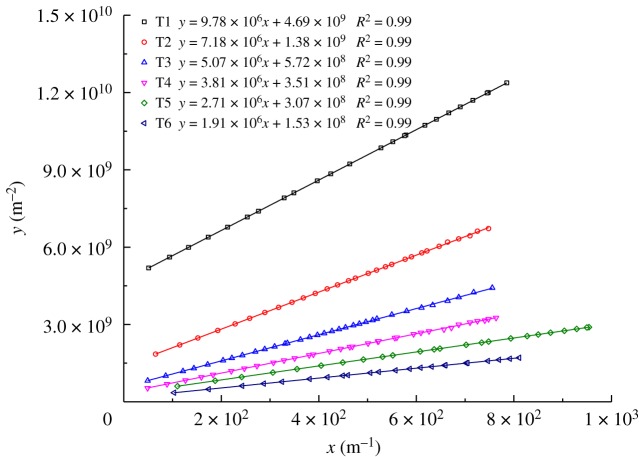
Relation between *y* and *x.*

The test results of the soil permeability parameters are shown in table 1. Figure 7 shows the determination of *k* and *β* in the gravel layers with different porosities. The results of *k* and *β* and the porosity of the gravel layers are shown in table 2.

**Table 1.** Permeability coefficient of the slope.

soil layer	silt	fine sand	gravel	silty clay	gravelly clay	bed rock
permeability (m^2^)	5.48 × 10^−12^	1.08 × 10^−11^	3.26 × 10^−9^	4.16 × 10^−13^	8.38 × 10^−13^	1.55 × 10^−12^

**Table 2.** Measured permeability and non-Darcy coefficient.

no.	porosity	permeability *k* (m^2^)	non-Darcy coefficient *β* (m^−1^)
T1	0.24	2.12 × 10^−10^	9.78 × 10^6^
T2	0.28	7.25 × 10^−10^	7.18 × 10^6^
T3	0.33	1.75 × 10^−9^	5.07 × 10^6^
T4	0.37	2.85 × 10^−9^	3.81 × 10^6^
T5	0.39	3.26 × 10^−9^	2.71 × 10^6^
T6	0.47	6.54 × 10^−9^	1.92 × 10^6^

In practical engineering, porosity is easier than permeability to obtain experimentally. The Kozney–Carman (*kc*) equation is one of the most famous formulae for liquid or gas flow in porous media and has been used to calculate the permeability of porous media worldwide for a long time. Eventually, the permeability could be summarized as a function of the particle diameter and porosity [20]. Permeability can be defined as

3.4 $$k=\frac{d^{2}\times n^{3}\times \phi_{s}^{2}}{150{\left(1-n\right)}^{2}},$$

where *d* is the particle diameter and *ϕ*_s_ is the shape coefficient.

**Figure 8.**
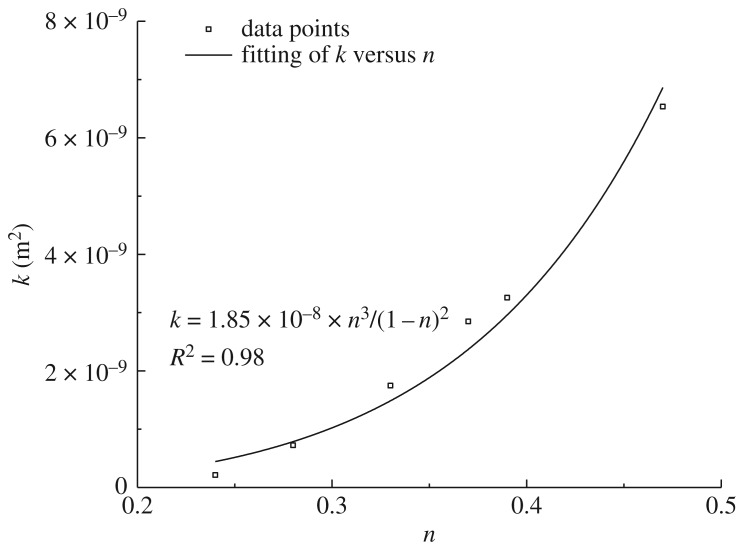
Relation between *k* and *n*.

In the gravel layers, the average size and shape coefficient of the particles are invariant; hence, *d* and *ϕ*_s_ may be considered constants. Then, the relationship between permeability and porosity can be expressed as follows:

3.5 $$k=a\frac{n^{3}}{{\left(1-n\right)}^{2}},$$

where *a* is constant (figure 8).

Through data fitting, we also present a new correlation:

3.6 $$k=1.85\times 10^{-8}\times \frac{n^{3}}{{\left(1-n\right)}^{2}}.$$

The correlation coefficient of equation (3.6) is 0.98; therefore, equation (3.6) is reasonable to use for practical applications in which the permeability of gravel layers needs to be determined.

The porosity, permeability and tortuosity are probably the most important factors determining the value of the non-Darcy coefficient. However, determining a realistic and precise tortuosity is not easy. Therefore, many scholars use only the porosity, *n*, and permeability, *k*, to characterize the non-Darcy coefficient, *β* [21,22], which can be written as

3.7 $$\beta =\frac{b}{{\left(kn\right)}^{c}},$$

where *b* and *c* are constants.

Taking the log of equation (3.7) leads to

3.8 log⁡β=lg⁡b−c log (kn).

**Figure 9.**
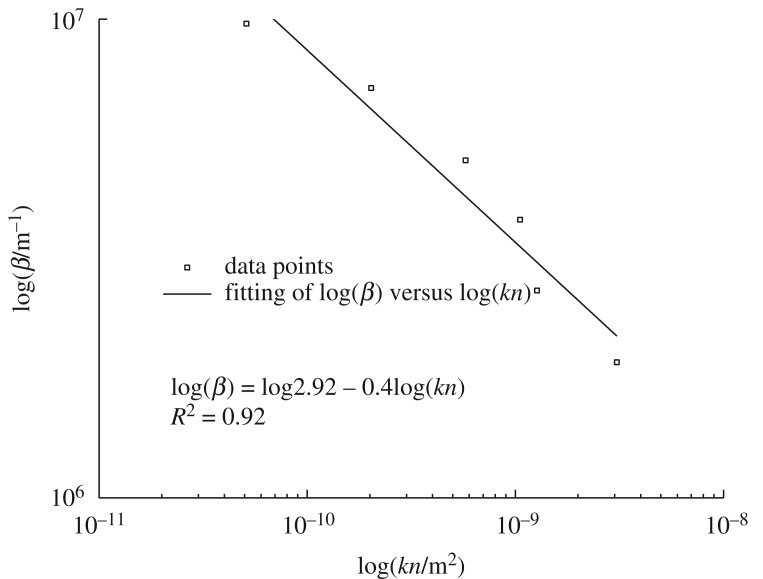
Relation between log(*β*) and log(*kn*).

From this straight line, *b* and *c* are determined, as shown in figure 9.

3.9 $$\beta =\frac{2.92}{{\left(kn\right)}^{0.4}}.$$

Using equation (3.6) to eliminate *k* in equation (3.9) gives

3.10 $$\beta =3.62\times 10^{3}\times \frac{{\left(1-n\right)}^{0.8}}{n^{1.6}}.$$

## 4. Model descriptions

### 4.1. Laminar flow in low-permeability aquifers

The groundwater flow in the silt layer, fine sand layer, silty clay layer, gravelly clay layer and bedrock is mainly driven by the water pressure. The seepage velocity is very slow, the inertia force is considerably less than the viscous drag, and the flow state of the groundwater is laminar flow. The relation between the fluid velocity and pressure gradient are linear, consistent with the Darcy law at the macroscale, and can be expressed as

4.1 $$v_{d}=-\frac{k_{d}}{\mu }(\nabla p_{d}-\rho g),$$

where the subscript *d* represents the Darcy seepage field.

Under unsteady-state conditions, the seepage continuity equation is

4.2 $$\rho \frac{\partial n_{d}}{\partial t}-\nabla \cdot \left(\frac{\rho k_{d}}{\mu }(\nabla p_{d}-\rho g)\right)=f_{d},$$

where *t* is the time, *f*_d_ is the source term, ∇ is the gradient operator and ∇⋅ is the divergence operator.

### 4.2. Forchheimer flow in the gravel layers

In the gravel layers, the flow state exhibits distinctly nonlinear behaviour and the relationship between the flow velocity and the pressure gradient closely obeys the Forchheimer equation, as shown through experimental verification. Under the precondition of incompressible flow, the equation of motion based on the Forchheimer equation can be written as

4.3 $$\rho c_{f}\frac{\partial v_{f}}{\partial t}+\beta_{f}\rho {v_{f}}^{2}+\frac{\mu }{k_{f}}v_{f}+\nabla p_{f}=f_{f},$$

and the continuity equation is

4.4 $$\frac{\partial (n_{f}\rho )}{\partial t}+\nabla \cdot (\rho v_{f})=f_{f},$$

where the subscript *f* represents the Forchheimer seepage fields, *c*_f_ is the acceleration coefficient and *f*_f_ is the flow mass force. In addition, the permeability *k*_f_ and the non-Darcy coefficient *β*_f_ of porous materials considerably affect the water flow through the media. The relation between the permeability and non-Darcy coefficient is described by equation (3.9).

### 4.3. Boundary conditions for the transition between two flow regions

During the whole process of groundwater flow in the slope, both the flow velocity and the pressure are continuous. Therefore, the transition boundary conditions can be defined as

4.5 $$p_{d}=p_{f}$$

and

4.6 $$v_{d}=v_{f}.$$

### 4.4. Numerical solution of the model

To establish the weak integral form of the water head, the Darcy equation and Forchheimer equation are rewritten in components in a Cartesian coordinate system and then equations (4.7) and (4.8) are combined to write equation (4.9):

4.7 $$\begin{array}{r} \frac{\partial u}{\partial x}+\frac{\partial v}{\partial y}=0, \end{array}$$

4.8 $$\begin{array}{r} H=Z+\frac{P}{\gamma }+\frac{v^{2}}{2g} \end{array}$$

4.9 $$\begin{array}{r} \;\text{and}\;\left[\frac{\partial H}{\partial x};\frac{\partial \delta H}{\partial x}\right]\times k_{x}+\left[\frac{\partial H}{\partial y};\frac{\partial \delta H}{\partial y}\right]\times k_{y}=0. \end{array}$$

The element birth and death method is used in the FELAC 2.2 software to solve the seepage flow problem with a free surface. A term is added to equation (4.9) so that when the water head is less than its position head, the left stiffness term becomes small by setting em = 1.0 ^−12^.

4.10 $$\left[\frac{\partial H}{\partial x};\frac{\partial \delta H}{\partial x}\right]\times k_{x}\times \text{vol}+\left[\frac{\partial H}{\partial y};\frac{\partial \delta H}{\partial y}\right]\times k_{y}\times \text{vol}+[H;H]\times \text{em}=0.$$

**Figure 10.**
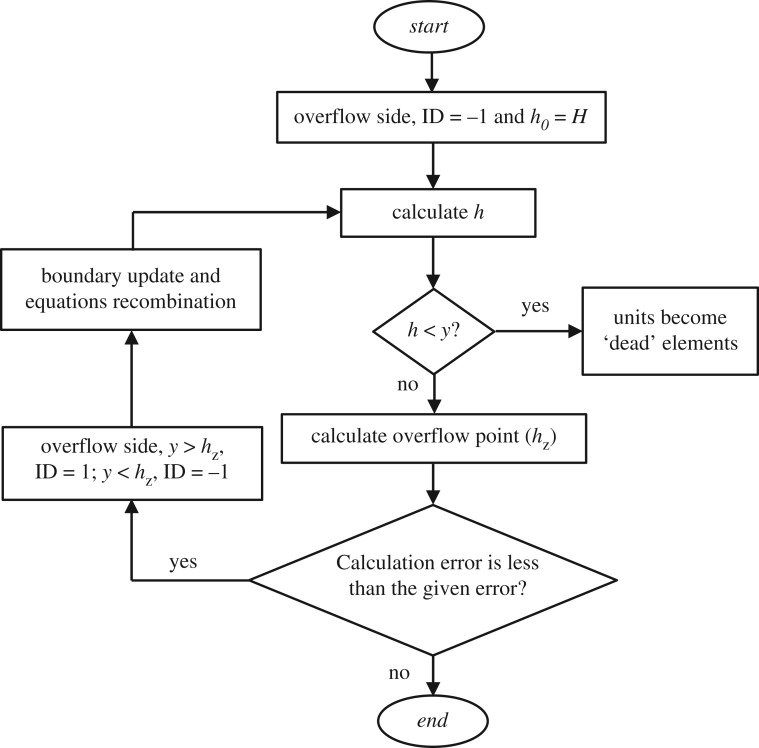
The entire iterative procedure to determine the free surface.

To use an iteration algorithm to calculate the free surface, setting an overflow point is necessary to balance the water head (*h*) with the position head (*H*). As the overflow point approaches, the boundary above the overflow point needs to be updated, and then the equations will be reorganized. Figure 10 illustrates the entire iterative procedure.

In addition, according to the principle of virtual work, the Darcy equation and Forchheimer equation are represented in the form of a weak integral. To solve the model, the finite-element method is combined with a finite volume method. Then, the numerical programs are developed with FELAC 2.2 software. The specific process of solving the model has been illustrated in detail in the literature [23].

### 4.5. Numerical model

**Figure 11.**
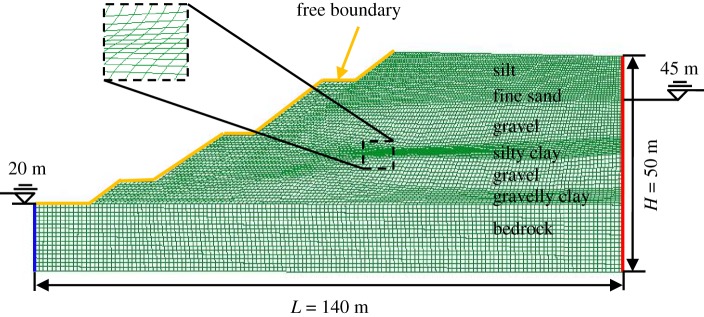
Numerical model of the cross-section N–N′.

According to the geologic section of the Yanshan Iron Mine (figure 2), a two-dimensional numerical model is established, as shown in figure 11. The length of the numerical model is 140 m, and its height is 50 m. The computing area is divided into 37 076 four-node quadrilateral elements. According to the hydrological conditions, the boundary conditions of the model are as follows. The constant water heads, *H* = 45 m and *H* = 20 m, are set at the right boundary (red boundary), which is near the Xinhe River, and the left boundary (blue boundary) of the slope, respectively. At the left boundary, the other slope sides are directly exposed to the atmosphere; therefore, they are free boundaries. At the interface of the Darcy seepage flow and Forchheimer seepage flow fields, we set *p*_d_ = *p*_f_ and *v*_d_ = *v*_f_. Newman boundary conditions with zero flux are applied to the rest of the external boundaries of the model. According to the hydrological conditions and laboratory tests, the hydrodynamic parameters were determined and are shown in table 1. For the sake of analysis, we are considering two options, as shown in table 3. In model 1, we use only the Darcy seepage equation to describe the entire flow field, while the computation of the Darcy equation is coupled with that of the Forchheimer equation in model 2.

**Table 3.** Projects of the numerical simulation.

model	calculation model
1	Darcy model (silt layer, fine sand layer, silty clay layer, gravel layers, gravelly clay layer and bedrock)
2	Darcy model (silt layer, fine sand layer, silty clay layer, gravelly clay layer and bedrock) + Forchheimer model (gravel layers)

## 5. Results and discussions

### 5.1. Numerical results

**Figure 12.**
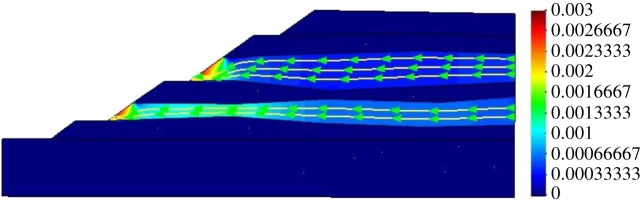
Distribution of the streamline and flow velocity (unit: m s^−1^).

**Figure 13.**
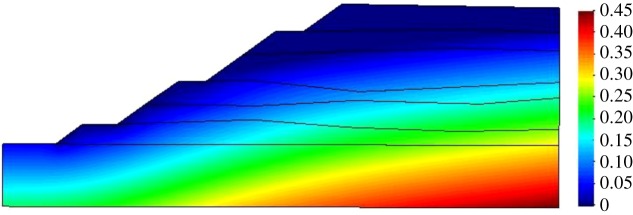
Distribution of the flow pressure during the water flow process (unit: MPa).

The velocity and pressure distribution of the water flow from the Xinhe River through the gravel layers to the open-pit mine are shown in figures 12 and 13. Figure 12 shows the stream function and velocity vector distribution when the fluid reaches a dynamic balance. It can be seen that the water velocity and pressure continuously vary. The water flows through the soil layers into the open-pit mine, and the gravel layers are the primary seepage channel. The velocity reaches a maximum value of 3 mm s^−1^ at the outlet of the slope surface; this value is approximately equal to the measured velocity of 2.3 mm s^−1^.

**Figure 14.**
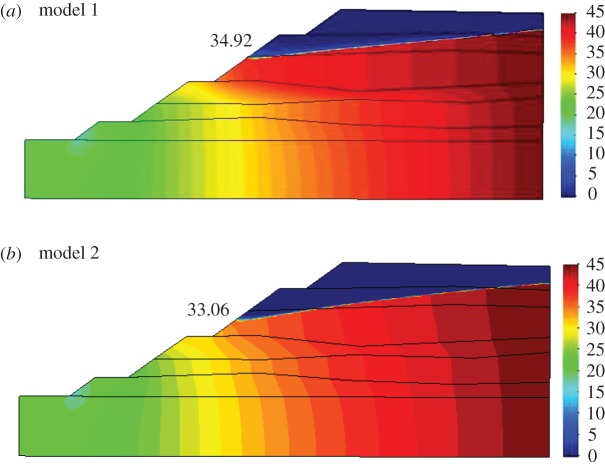
(*a,b*) Numerical results of water surfaces (unit: m).

**Figure 15.**
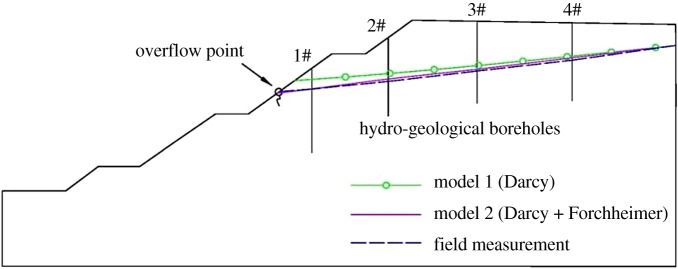
Comparisons between calculation result and field observation results.

To verify the calculated feasibility and accuracy of the simulation results, two different models are used to calculate the same scenario; the results are very different and are shown in figures 14 and 15. The height of the overflow points are 34.92 and 33.06 m, respectively. The free surface calculated by using model 2 is in good agreement with the *in situ* measurement, and the location of the overflow point is consistent with the field observations (figure 3*c*); however, there is an evident deviation of the simulation results from the measured data when model 1 is used. Based on the theoretical analysis, according to the comparison of the Darcy equation and Forchheimer equation, the viscous resistance and inertial resistance are both included in the Forchheimer equation and have the same order of magnitude. Additionally, the position of the viscous resistance and inertial resistance are both important. The pressure loss is caused by the combination of the viscous resistance and inertial resistance, and more rapid decreases in pressure are observed when using the Forchheimer equation than when using the Darcy equation. Therefore, the results of the Darcy model are rather high.

### 5.2. Further discussion

As the water burst into the open pit, the permeability of the gravel layers increases with the loss of small particles due to the high-velocity water flow. Under certain water pressure conditions, the permeability and non-Darcy factor of the gravel layers not only directly affect the viscous drag and inertia resistance but also control the distribution of fluid pressure in the slope, the location of the free surface and the amount of water inflow. In addition, there is a one-to-one relationship between the permeability and non-Darcy factor [17,24]. Therefore, a sensitivity analysis of the permeability of the gravel layers is important to guide the accurate prediction of the amount of water inflow and the location of the free surface.

#### 5.2.1. The effect of porosity on the overflow point

Determination of the seepage overflow point is very important in the analysis of soil-slope stabilization problems. To study the effect of the permeability of the gravel layers on the overflow point location, five cases are considered. Since some of the small particles are lost, the following five situations are considered: the porosity of the gravel layers are 0.1, 0.2, 0.3, 0.4 and 0.5, and the corresponding permeabilities are 2.28 × 10^−11^, 2.31 × 10^−10^, 1.02 × 10^−9^, 3.29 × 10^−9^ and 9.25 × 10^−9^ m^2^, respectively (calculated by using equation (3.6)). The non-Darcy factor, *β*, is calculated by equation (3.10). In addition, the permeability of the other layers remains unchanged. Table 4 shows the various seepage field physical quantities influenced by the values of *n*.

**Table 4.** Various physical quantities in the seepage field of the gravel layers that are influenced by the values of *n*.

*n*	*k*/(m^2^)	*β*/(m^−1^)	model	maximum seepage gradient	maximum seepage gradient increment (%)	overflow point height (m)	overflow point height increment (%)
0.1	2.28 × 10^−11^	1.32 × 10^5^	1	0.28	—	32.64	—
			2	0.30	7.14	31.88	−2.33
0.2	2.31 × 10^−10^	3.98 × 10^4^	1	0.24	—	33.63	—
			2	0.26	8.33	32.31	−3.93
0.3	1.02 × 10^−9^	1.87 × 10^4^	1	0.19	—	34.35	—
			2	0.21	10.53	32.74	−4.69
0.4	3.29 × 10^−9^	1.04 × 10^4^	1	0.13	—	34.97	—
			2	0.15	15.38	33.09	−5.38
0.5	9.25 × 10^−9^	6.30 × 10^3^	1	0.11	—	35.71	—
			2	0.13	18.19	33.42	−6.41

When the permeability of the gravel layers is low, the nonlinear phenomenon of the seepage flow is unclear, and the calculation results from both models are very similar. The height of the overflow point is not very different between the two models, therefore, the error of model 1 is relatively small. With an increase in the permeability, the height of the overflow points of model 1 and model 2 gradually increase. Additionally, the discrepancy between the two results increases, and the height of the overflow point calculated by model 2 is consistently lower than that of model 1. When the permeability is higher, the water line is relatively gentle, while the relative curvature of the water level is greater due to the low permeability. In the seepage outlet area, the increase in the hydraulic gradient is obvious. Accordingly, it is necessary to calculate the free surface with model 2 to simulate large pores and high permeability. The phenomenon of non-Darcy seepage is less apparent when the flow is through less permeable porous media. However, the high permeability layers and low-permeability layers are mixed together in the natural geological environment or slope, complicating the seepage field. To more objectively and accurately determine the location of the overflow point, it is necessary to use a higher-precision calculation method, especially in the areas where the flow velocity is rapidly changing and in the areas that need to be studied in slope engineering.

#### 5.2.2. The effect of porosity on the water inflow

**Figure 16.**
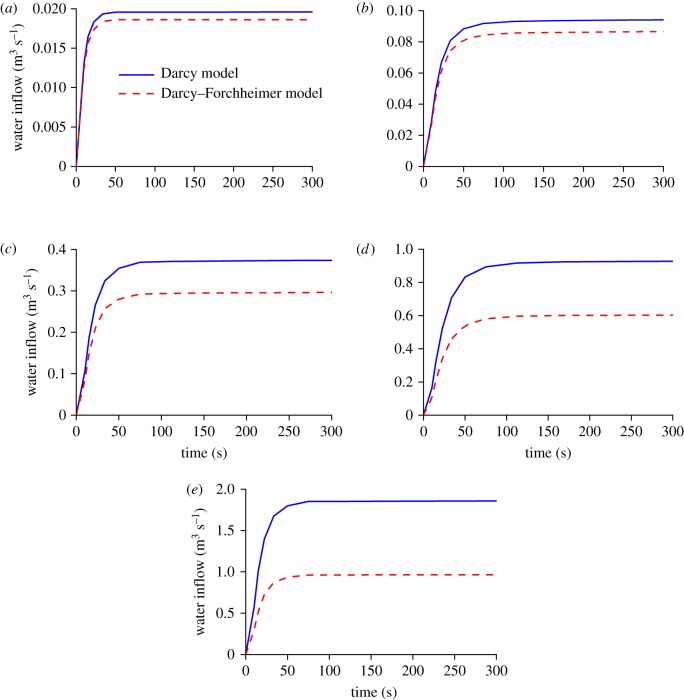
Change in the water inflow with time under different permeabilities: (*a*) 2.28 × 10^−11^ m^2^, (*b*) 2.31 × 10^−10^ m^2^, (*c*) 1.02 × 10^−9^ m^2^, (*d*) 3.29 × 10^−9^ m^2^ and (*e*) 9.25 × 10^−9^ m^2.^

The relation curves that describe the variation in water inflow with time are presented in figure 16. The results show that the change in the amount of water inflow can be divided into three stages: (1) accelerated growth; (2) slow growth; and (3) steady flow. In the accelerated growth period, the rate of increase in the water inflow calculated by using the two models are fairly similar under the condition of low permeability, but they differ considerably with greater permeability, and the rates of the increase in the water inflow calculated by using model 1 is greater than that of model 2. The higher the permeability is, the greater the difference between the two model results. In addition, the amount of water inflow can become stable, even within a very short time, because in most cases, the transient fluid flow through the porous media decays very quickly, and the time derivative terms of equations (4.2)–(4.4) also decrease quickly, transforming the transient equations into steady-state equations [25].

According to our analysis, the results obtained through the two models were found to have few discrepancies in the steady flow period under the condition of low permeability. With the gradual increase in permeability, the maximum water inflow increases gradually and the maximum water inflow calculated by model 1 is consistently greater than that of model 2. That result is because the Forchheimer equation includes the square of the water velocity, considerably slower than the water velocity used in the Darcy equation under the same condition.

**Figure 17.**
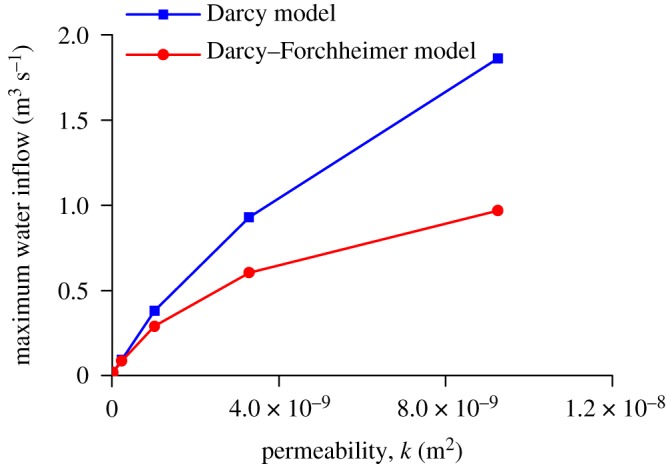
Change in the maximum water inflow with permeability.

Figure 17 shows that the maximum water inflow varies with permeability. Under certain boundary and initial conditions, the maximum water inflow increases with the increase in permeability, while the maximum water inflow of model 2 increases more slowly than that of model 1. Thus, it is necessary to adopt model 2 to predict the maximum water inflow for the open-pit slope that contains the high-permeability gravel layers. If we use model 1 to calculate the maximum water inflow, the results would greatly deviate from the actual situation. As the permeability increases, the difference between these two models increases.

## 6. Conclusion

The permeability of soil layers is obtained according to laboratory seepage experiments. The functions of the permeability versus porosity and non-Darcy factor versus porosity and the relationship among them were determined. A coupling model is established to study the water flow behaviour in the Yanshan Iron Mine. The effect of porosity on the overflow point and water inflow was discussed. The main conclusions are drawn as follows:

1. Laboratory tests show that the seepage behaviour in the silt layer, fine sand layer, silty clay layer and gravelly clay layer follow the traditional Darcy law, while the gravel layers show clear nonlinear characteristics. The permeability increases exponentially, and the non-Darcy coefficient decreases exponentially with an increase in the porosity of the gravel layers, and the relations among the permeability, the porosity and the non-Darcy coefficient are established as equations to use in future engineering applications.
2. For a slope containing gravel layers with high permeability, considering the seepage in the slope during the transition from Darcy flow to Forchheimer flow, a coupled mathematical model that considers both of these flow fields is established with Darcy flow in the low-permeability layers and Forchheimer flow in the high-permeability layers. This method provides a model base for predicting the water inflow and computing the free surface of a slope.
3. This model is used in the seepage analysis of a cross section of the eastern slope of the Yanshan Iron Mine. By comparing the two solutions from the Darcy model and the Darcy–Forchheimer model, the results indicate that the free surface calculated by the Darcy–Forchheimer model is in good agreement with the *in situ* measurements; however, there is an evident deviation of the simulation results from the measured data when the Darcy model is used. Therefore, the model is suitable for use in high-permeability slope engineering.
4. The sensitivity analysis of the gravel layers indicates that with the increase in the permeability of the gravel layers, the water inflow increases, and the location of the overflow point moves up the slope. The results of the Darcy model are greater than those of the Darcy–Forchheimer model, and the greater the permeability is, the greater the difference between the two models. Accordingly, it is necessary to calculate the free surface and water inflow with the Darcy–Forchheimer model when the slope includes large pores and high permeability.
5. In slope engineering, although the calculation results of the Darcy model are more conservative in terms of safety, the precision and objectivity of the description of the seepage behaviours can be improved by applying the Darcy–Forchheimer model. In mine drainage engineering, the more accurate the prediction of water inflow is, the lower the economic cost.

## Data accessibility

All original data are deposited at the Dryad Digital Repository: http://dx.doi.org/10.5061/dryad.qb4b7 [26].
